# Biased antagonism of a series of bicyclic CXCR2 intracellular allosteric modulators

**DOI:** 10.3389/fphar.2025.1631129

**Published:** 2025-07-14

**Authors:** Brent Van Bosstraeten, Katrijn Boon, Max Van Hoof, Wim Dehaen, Martyna Szpakowska, Andy Chevigné, Dominique Schols, Steven De Jonghe, Tom Van Loy

**Affiliations:** ^1^ Department of Microbiology, Immunology and Transplantation, Rega Institute for Medical Research, Molecular, Structural and Translational Virology Research Group, KU Leuven, Leuven, Belgium; ^2^ Department of Chemistry, KU Leuven, Sustainable Chemistry for Metals and Molecules, Leuven, Belgium; ^3^ Department of Infection and Immunity, Immuno-Pharmacology and Interactomics, Luxembourg Institute of Health (LIH), Esch-surAlzette, Luxembourg

**Keywords:** CXCR2, antagonist, bias, G protein, G protein-coupled receptor, NanoBRET

## Abstract

Targeting the human chemokine receptor (CXCR2) holds significant potential in treating inflammatory diseases and cancer. In this study, we investigate the biased properties of previously reported CXCR2 antagonists (i.e., the MVH compounds). These antagonists likely bind to a conserved intracellular pocket that is also targeted by the well-known CXCR2 antagonist, navarixin. However, unlike navarixin, the MVH compounds are derived from a completely distinct chemotype, raising the possibility that they may engage the receptor differently and produce biased inhibition of downstream signaling pathways. To deduce these potential biased properties, the compounds were investigated using two NanoBRET-based assays, showing a preferential inhibition of CXCR2-mediated β-arrestin recruitment over G protein activation. Furthermore, a detailed statistical analysis revealed an additional bias in the inhibition profiles dependent on the specific ELR+ chemokine used to stimulate the receptor. Altogether, these results describe the MVH compounds as the first set of biased CXCR2 intracellular antagonists.

## Introduction

The human chemokine receptor (CXCR2) is a G protein-coupled receptor (GPCR) that gained the attention of the drug discovery community due to its promise as a target for the treatment of various inflammatory disorders and different types of cancer (Ha et al., 2017; Poeta et al., 2019; Qiao et al., 2017; Cheng et al., 2019). CXCR2 is particularly involved in neutrophil recruitment, tumor progression, and the establishment of a pre-metastatic niche, which makes it a popular target for diseases such as chronic obstructive pulmonary disease (COPD), rheumatoid arthritis, asthma, and cancer (Ha et al., 2017; Cheng et al., 2019). To discover novel CXCR2 antagonists, we recently applied a scaffold hopping strategy starting from the thiazolo[4,5-*d*]pyrimidine-based CXCR2 antagonist **AZ-8309**. It led to the discovery of several CXCR2 antagonists, based on unexplored chemotypes (Van Hoof et al., 2022) (Supplementary Table S1), which are assumed to occupy an intracellular allosteric binding pocket that is conserved between several human chemokine receptors (Nicholls et al., 2008; Salchow et al., 2010).

Upon activation, CXCR2, like most GPCRs, initiates signal transduction through heterotrimeric G protein pathways, whereas β-arrestins primarily contribute to receptor desensitization and endocytosis, and may modulate signaling through scaffolding functions (Kolb et al., 2022). These pathways can lead to distinct physiological and pathophysiological outcomes (Cheng et al., 2019; Kolb et al., 2022). An emerging theme in GPCR research is the concept of biased signaling. This was initially proposed in the context of the endogenous ligands of GPCRs, in which different ligands (agonists) for the same receptor can preferentially activate or block specific and distinct downstream signaling pathways over others (Kolb et al., 2022). Later on, biased small-molecule agonists were synthesized for many different GPCRs, whereas the field of biased antagonists is lagging behind (Rankovic et al., 2016; Kelly et al., 2023; Liu et al., 2021).

Conventional GPCR antagonists are often identified based on their ability to inhibit the activity of a natural agonist using functional GPCR assays with a single readout, such as second messenger responses (e.g., calcium mobilization or cAMP modulation), without assessing the antagonist’s potential to differentially inhibit multiple receptor-mediated pathways. As a result, it often remains unexplored whether antagonists are “balanced” (i.e., inhibiting multiple signaling pathways equally) or “biased” (i.e., preferentially inhibiting particular signaling pathways over others). Although for most GPCRs, including CXCR2, there are currently no indications that biased agonists or antagonists may be endowed with beneficial clinical properties, it is conceptualized that they might have improved efficacy or a better safety profile as they target disease-related pathways while leaving other pathways mediated by the same GPCR unaffected (Fan and Wang, 2025). Therefore, it presents an interesting concept that warrants further investigation. An essential requirement for this is the design, synthesis, and characterization of biased small-molecule GPCR modulators that can be used as research tools to investigate their potential beneficial properties.

Previously, we determined the CXCR2 antagonism of a series of compounds *via* a calcium mobilization assay (Van Hoof et al., 2023). These compounds bind to a conserved intracellular pocket on the chemokine receptor, functioning as allosteric modulators (Van Hoof et al., 2022; Van Hoof et al., 2023). Interestingly, the well-known potent CXCR2 antagonist navarixin, which represents a chemically distinct scaffold, also targets identical intracellular receptor sites. Given that both chemotypes engage the same binding pocket, yet differ structurally, we hypothesized that they may exhibit different properties. In this study, we employed NanoBRET-based assays that measure heterotrimeric G protein dissociation and β-arrestin recruitment, enabling the identification of possible biased properties of the previously reported CXCR2 antagonists (Boon et al., 2023; Luís et al., 2022).

## Materials and methods

### Chemokines, reagents, and plasmids

Recombinant human CXCL1–3 and CXCL5–8 were obtained from PeproTech. hCXCR2 expression plasmid (pUNO1-kIL08RB) was purchased from InvivoGen. Poly-D-lysine (PDL; #2780) was purchased from MERCK. Nano-Glo^®^ Vivazine™ substrate (#N2581) was purchased from Promega. The REGA-SIGN plasmids and the NanoLux plasmids (i.e., CXCR2.mNeongreen (mNG), CXCR2.Nanoluciferase (NLuc), and β-arrestin1.Nluc) were previously described (Boon et al., 2023; Luís et al., 2022). The compounds were dissolved in DMSO.

### Cell lines

Human embryonic kidney cells (HEK293A, American Type Culture Collection (ATCC)) were cultured in Dulbecco’s modified Eagle medium, high glucose (DMEM; #41965, Thermo Fisher Scientific), supplemented with 10% fetal bovine serum (FBS; #SV30160.03, Cytiva), which is referred to as growth medium from here onward. HEK293A cells stably expressing hCXCR2 were transfected with pcDNA3.1(+) hCXCR2 plasmid using FuGENE HD transfection reagent (Promega) according to the manufacturer’s protocol. HEK293A.hCXCR2 cells were cultured in the growth medium supplemented with 500 μg/mL geneticin. Receptors’ expression was validated by flow cytometry, as described previously (Boon et al., 2024).

### NanoBRET cell transfection and seeding

Cells were trypsinized and resuspended at 3 × 10^5^ cell/mL in the growth medium, whereafter they were incubated for 2 hours at RT. Cells were transfected in suspension using FuGENE^®^ HD Transfection Reagent at a 3:1 reagent to DNA ratio in Opti-MEM™ I Reduced-Serum Medium, with the final DNA concentration of 1 μg/μL. The transfection mixture was incubated for 10 min at RT before adding it to the cell suspension. Transfected cells were seeded at 3.0 × 10^4^ cells/well in a white, clear, flat-bottom 96-well plate coated with 100 μg/mL poly-D-lysin (P7280, Sigma Aldrich) and incubated for 48 h at 37°C and 5% CO_2_.

### NanoBRET-based G protein activation inhibition assay

HEK293A.CXCR2 cells were transiently co-transfected with plasmids encoding Gα_i1_ protein tagged with nanoluciferase (NLuc, donor) and Gγ_2_ protein tagged with LSS-mKATE2 acceptor in a 1:10 donor to acceptor ratio, as described by Boon et al. (2023). Following the 48-h incubation period, cells were washed with the assay buffer. Compounds were serially diluted in 1:500 Nano-Glo^®^ Vivazine^™^ working solution (#N2581, Promega), and 90 µL of compound/Vivazine mix was added, followed by incubation for 45 min at 37°C and 5% CO_2_. Plates were transferred to the FLIPR Penta system (Molecular Devices) at 37°C, and all steps onward are automated and performed by the FLIPR Penta system. After a 10-min equilibration period, the baseline BRET signal was determined by five consecutive reads followed by automatic administration of 10 µL of 10× ligand (Table 1) to the cell plate. Changes in the BRET signal was measured for 10 min, with each read spaced by 2.5 s. Measurements were acquired using a 440- to 480-nm donor emission filter (#0200-6179, Molecular Devices) and a custom 615 nm AT600lp acceptor emission filter (#296420, Chroma).

**TABLE 1:** Overview of EC_80_ values of CXCR2 ligands used in NanoBRET assays (Boon et al., 2024).

	CXCL1	CXCL2	CXCL3	CXCL5	CXCL6	CXCL7	CXCL8
G-protein EC_80_ (nM)	3.20	2.15	8.92	5.16	17.12	4.09	4.16
β-arrestin EC_80_ (nM)	59.18	147.8	100.10	86.19	168.00	100.70	17.29

### NanoBRET β-arrestin1 recruitment inhibition assay

HEK293A cells were transiently co-transfected with plasmids encoding β-arrestin1 protein tagged with NLuc (donor) and CXCR2 protein tagged with mNeonGreen (mNG) acceptor in a 1:10 donor to acceptor ratio, as described previously (Luís et al., 2022). All subsequent steps are identical to those in “NanoBRET-based G protein activation inhibition assay,” which are described above. Measurements were acquired using a 440- to 480-nm donor emission filter (#0200-6179, Molecular Devices) and a 515- to 575-nm acceptor emission filter (#0200-6203, Molecular Devices)

### NanoBRET analysis and bias calculations

BRET ratios were extracted from ScreenWorks software (Molecular Devices) and represent the ratio between the acceptor emission of LSS-mKATE2 or mNG (G protein or β-arrestin recruitment, respectively) and NLuc donor emission. The basal BRET ratio was defined as the average BRET ratio of the five consecutive readings preceding ligand addition. Changes in BRET values post ligand administration (∆BRET) were calculated for each well as a percentage difference to the previously defined basal BRET ratio. Average ∆BRET values were baseline corrected by subtracting the average negative control BRET ratio (unstimulated condition). Subsequently, the negative area under the curve (AUC_neg_) was used as the readout for G protein activation, whereas positive area under the curve (AUC_pos_) was used as the readout for β-arrestin recruitment (**calculation 1**). AUCs were normalized to the positive control (stimulated condition); whereafter, dose–response curves were fitted to log (inhibitor) vs response (three parameters) in GraphPad 10.2.0 (GraphPad Software). Bottom values were constrained to 0 and top values were constrained to 100 in REGA-SIGN and β-arrestin recruitment to extract the IC_50_ value.

Calculation 1: calculation of the BRET signal.

1. $BRET\;ratio=\frac{{615nm}_{em}}{{460nm}_{em}}.$

2. $∆BRET=\left[\frac{{BRET}_{stim}-{BRET}_{basal}}{{BRET}_{basal}}\right]\times 100.$

3. $NC\;corrected∆BRET={∆BRET}_{\exp}-{mean∆BRET}_{NC}.$

4. a. Neg AUC of NC corrected∆BRET=Gα activation read out. b. Pos AUC of NC corrected∆BRET=β−arrestin recruitment.

For signaling bias calculations, constraints were set to bottom values equal to zero. The calculated top values generated by nonlinear fit log (inhibitor) vs response (three parameters) in GraphPad 10.2.1 were taken as I_max_. I_max_ values were normalized to I_max_ of navarixin, which was set at 100%. The bias index values were calculated, as is shown in **calculation 2**. Briefly, log(I_max_/IC_50_) was calculated. Second, for each pathway, ∆log(I_max_/IC_50_) values are calculated by subtracting the log(I_max_/IC_50_) of the reference compound (i.e., navarixin) from the log(I_max_/IC_50_) of each compound. Finally, the bias index was calculated by subtracting the ∆log (I_max_/IC_50_) of the tested compound from the stated reference pathway from that of the pathway of interest. Notably, when I_max_ and IC_50_ could not be determined due to low activation, log(I_max_/IC_50_) was taken as 0. The statistical significance of H1 was evaluated using a one-sample T-test with Benjamini-Hochberg correction applied for multiple comparisons, whereas H2 was assessed using a one-way ANOVA followed by Tukey’s test.

Calculation 2: calculation of the bias index.

1. $\log{\left(\frac{I_{\max}}{{IC}_{50}}\right)}_{compound}.$

2. $∆\log\left(\frac{I_{\max}}{{IC}_{50}}\right)=\log{\left(\frac{I_{\max}}{{IC}_{50}}\right)}_{compound}-\log{\left(\frac{I_{\max}}{{IC}_{50}}\right)}_{ref:Navarixin}.$

3. $Bias\;index={∆\mathrm{Log}\left(\frac{I_{\max}}{{IC}_{50}}\right)}_{P1:\beta -arr1}-{∆\mathrm{Log}\left(\frac{I_{\max}}{{IC}_{50}}\right)}_{P2:Gai1\;}.$

### Bias plot visualization

Bias plots are equimolar comparisons between the two pathways of interest. They are visualized by setting out the normalized concentration–response curve (normalized using the I_max_ of navarixin as 100%) of one pathway against the other.

## Results

### Navarixin inhibits ELR+ chemokine-induced CXCR2 Gα_i1_ protein activation and β-arrestin1 recruitment equally, and thus, it presents as an unbiased reference compound for CXCR2 antagonism

Analysis of the biased inhibitory properties of small-molecule antagonists critically relies on the existence of an unbiased reference antagonist, to which the activity of the test compounds can be compared. We hypothesized that navarixin could serve as such an unbiased reference antagonist for CXCR2. To validate this, we assessed its ability to inhibit CXCR2-induced G protein dissociation and β-arrestin recruitment, respectively, using previously established NanoBRET-based assays. For the analysis of the inhibition of G protein activation, dissociation of the relevant G protein subtype Gα_i1_ was quantified using the REGA-SIGN G protein biosensor assay (Boon et al., 2023). To quantify the inhibition of β-arrestin recruitment, we analyzed β-arrestin1 (βarr1) recruitment using the NanoBRET-based NanoLux assay (Luís et al., 2022). Although the CXCL8–CXCR2 signaling axis is the best characterized one, all ELR+ chemokines (CXCL1–3 and CXCL5–8) were included as agonists in both assays as they all activate CXCR2 and are associated with distinct pathophysiological functions (Cheng et al., 2019).

Briefly, HEK293A.CXCR2 or HEK293A cells were transfected with Gα_i1_-91-NLuc and Gγ2-LSSmKATE2 (REGA-SIGN biosensor pair) or β-arrestin1.mNG and CXCR2.NLuc (NanoLux biosensor pair) encoding plasmids, respectively. The cells were subsequently incubated with the compound, which was serially diluted using an identical concentration range (15–0.021 µM) for 45 min. Finally, cells were stimulated with the different ELR+ chemokines (at their previously determined EC_80_ value) for Gα_i1_ or β-arrestin1 activation, respectively (Boon et al., 2024). The percentage inhibition was then calculated, and concentration–response curves were generated to determine the inhibitory efficacy (I_max_) and potency (IC_50_) values, which are shown in Table 2.

**TABLE 2:** Inhibition efficacy (I_max_) and potency (IC_50_) of selected compounds.

Compound ID		CXCL1		CXCL2		CXCL3		CXCL5		CXCL6		CXCL7		CXCL8	
		Gαi1	βarr1	Gαi1	βarr1	Gαi1	βarr1	Gαi1	βarr1	Gαi1	βarr1	Gαi1	βarr1	Gαi1	βarr1
Navarixin	IC_50_ µM	0.08 ± 0.24	0.05 ± 0.05	0.15 ± 0.24	0.12 ± 0.06	0.13 ± 0.24	0.07 ± 0.09	0.01 ± 0.24	0.01 ± 0.01	0.11 ± 0.24	0.06 ± 0.05	0.14 ± 0.24	0.11 ± 0.07	0.71 ± 0.24	0.13 ± 0.03
	I_max_ %	100	100	100	100	100	100	100	100	100	100	100	100	100	100
AZ10397767	IC_50_ µM	0.43 ± 0.24	0.15 ± 0.05	0.30 ± 0.24	0.08 ± 0.02	1.44 ± 0.24	0.07 ± 0.04	0.58 ± 0.24	0.05 ± 0.05	0.19 ± 0.24	0.03 ± 0.01	0.25 ± 0.24	0.02 ± 0.00	0.02 ± 0.24	0.16 ± 0.24
	I_max_ %	100	100	100	100	98.8	100	95.7	100	100	100	100	100	98.5	100
MVH-3	IC_50_ µM	>15.00	0.98 ± 0.18	6.24 ± 1.15	0.58 ± 0.17	>15.00	1.59 ± 0.43	10.23 ± 6.52	0.84 ± 0.16	7.53 ± 1.74	0.70 ± 0.17	10.90 ± 9.58	0.16 ± 0.05	>15.00	1.34 ± 0.45
	I_max_ %	<50	100	100	100	<50	100	93.7 ± 5.53	100	<50	100	<50	95.75 ± 3.49	<50	90.8 ± 4.62
MVH-9	IC_50_ µM	2.95 ± 1.41	0.15 ± 0.06	0.68 ± 0.22	0.07 ± 0.01	4.72 ± 1.94	0.17 ± 0.05	0.71 ± 0.38	0.07 ± 0.00	1.08 ± 1.00	0.07 ± 0.02	1.36 ± 1.23	0.03 ± 0.01	3.75 ± 1.72	0.15 ± 0.03
	I_max_ %	100	100	100	100	100	100	100	100	100	100	100	100	100	100
MVH-15	IC_50_ µM	>15.00	3.51 ± 0.83	>15.00	2.06 ± 0.57	>15.00	4.29 ± 1.16	>15.00	2.10 ± 0.36	>15.00	2.28 ± 0.57	>15.00	0.70 ± 0.14	>15.00	4.07 ± 1.60
	I_max_ %	<50	100	<50	100	<50	100	<50	100	<50	100	<50	100	<50	100
MVH-22	IC_50_ µM	>15.00	2.85 ± 0.68	>15.00	1.50 ± 0.18	>15.00	3.44 ± 0.75	>15.00	1.96 ± 0.53	>15.00	1.47 ± 0.20	>15.00	0.50 ± 0.07	>15.00	2.66 ± 0.30
	I_max_ %	<50	100	<50	100	<50	100	<50	100	<50	100	<50	97.6 ± 3.45	<50	100
MVH-23	IC_50_ µM	1.00 ± 0.30	0.06 ± 0.01	0.35 ± 0.22	0.05 ± 0.02	1.93 ± 0.30	0.08 ± 0.01	0.40 ± 0.15	0.05 ± 0.02	0.58 ± 0.65	0.03 ± 0.01	0.32 ± 0.12	0.02 ± 0.01	1.41 ± 0.25	0.09 ± 0.03
	I_max_ %	94.85 ± 3.70	100	100	100	100	100	94.29 ± 2.32	100	100	100	100	100	100	100
MVH-24	IC_50_ µM	2.08 ± 0.42	0.17 ± 0.06	1.21 ± 0.63	0.08 ± 0.04	14.79 ± 10.14	0.14 ± 0.05	1.43 ± 0.71	0.09 ± 0.03	1.06 ± 0.75	0.07 ± 0.02	0.75 ± 0.26	0.03 ± 0.01	2.49 ± 1.24	0.15 ± 0.04
	I_max_ %	96.47 ± 3.69	100	100	100	100	99.42 ± 0.85	98.52 ± 2.04	100	100	100	94.94 ± 3.57	100	100	100
MVH-30	IC_50_ µM	>15.00	4.45 ± 2.05	>15.00	2.41 ± 0.54	>15.00	4.50 ± 0.87	>15.00	3.24 ± 0.97	>15.00	1.91 ± 0.78	>15.00	0.75 ± 0.09	>15.00	3.50 ± 1.77
	I_max_ %	<50	100	<50	100	<50	100	<50	100	<50	100	<50	96.73 ± 0.86	<50	100
MVH-32	IC_50_ µM	5.95 ± 2.64	0.28 ± 0.11	2.98 ± 0.82	0.13 ± 0.04	>15.00	0.27 ± 0.07	3.09 ± 1.12	0.17 ± 0.03	3.18 ± 0.72	0.14 ± 0.09	2.22 ± 0.40	0.05 ± 0.01	3.57 ± 2.10	0.19 ± 0.11
	I_max_ %	100	100	100	100	<50	100	100	100	100	100	100	100	99.76 ± 2.64	100
MVH-33	IC_50_ µM	>15.00	1.46 ± 0.58	>15.00	0.66 ± 0.21	>15.00	1.53 ± 0.26	13.07 ± 4.76	1.12 ± 0.26	>15.00	0.79 ± 0.37	10.11 ± 4.80	0.34 ± 0.13	>15.00	1.51 ± 1.01
	I_max_ %	<50	98.15 ± 4.28	<50	92.98 ± 2.58	<50	96.91 ± 2.10	100	100	<50	93.68 ± 7.03	98.43 ± 2.62	100	<50	100
MVH-35	IC_50_ µM	>15.00	3.13 ± 1.17	>15.00	2.53 ± 0.73	>15.00	3.20 ± 2.78	>15.00	3.33 ± 0.92	>15.00	2.81 ± 0.33	>15.00	1.00 ± 0.54	>15.00	3.87 ± 1.80
	I_max_ %	<50	92.88 ± 3.12	<50	100	<50	100	<50	100	<50	100	<50	96.07 ± 5.83	<50	97.60 ± 5.36
MVH-46	IC_50_ µM	14.05 ± 14.32	0.48 ± 0.09	2.6 ± 1.30	0.25 ± 0.08	>15.00	0.87 ± 0.52	6.50 ± 7.08	0.32 ± 0.12	2.71 ± 1.87	0.38 ± 0.11	3.47 ± 1.88	0.09 ± 0.02	12.86 ± 4.81	0.48 ± 0.06
	I_max_ %	100	100	100	100	<50	100	100	100	100	100	100	100	100	100
MVH-52	IC_50_ µM	>15.00	1.51 ± 0.52	12.46 ± 2.98	0.68 ± 0.23	>15.00	1.33 ± 0.91	>15.00	0.89 ± 0.34	>15.00	0.67 ± 0.17	13.18 ± 7.27	0.25 ± 0.09	>15.00	1.80 ± 0.38
	I_max_ %	<50	93.93 ± 3.60	100	99.75 ± 5.45	<50	99.22 ± 2.04	<50	100	<50	99.98 ± 2.53	100	96.94 ± 4.36	<50	100
MVH-61	IC_50_ µM	11.09 ± 2.00	1.01 ± 0.21	>15.00	0.42 ± 0.22	>15.00	1.29 ± 0.39	>15.00	0.82 ± 0.54	>15.00	0.49 ± 0.14	5.24 ± 0.70	0.19 ± 0.09	>15.00	1.10 ± 0.41
	I_max_ %	98.96 ± 3.66	92.48 ± 1.67	<50	93.93 ± 5.71	<50	100	<50	100	<50	100	100	100	<50	99.58 ± 8.44
MVH-81	IC_50_ µM	>15.00	2.36 ± 0.67	>15.00	0.80 ± 0.19	>15.00	2.67 ± 0.63	>15.00	1.18 ± 0.35	>15.00	1.00 ± 0.27	13.87 ± 4.40	0.39 ± 0.20	>15.00	2.25 ± 0.79
	I_max_ %	<50	99.94 ± 2.94	<50	99.18 ± 2.21	<50	100	<50	100	<50	99.41± 2.50	100	100	<50	100
MVH-82	IC_50_ µM	>15.00	2.13 ± 0.34	>15.00	0.74 ± 0.16	>15.00	2.53 ± 0.15	>15.00	1.33 ± 0.47	>15.00	0.88 ± 0.47	>15.00	0.37 ± 0.11	>15.00	2.32 ± 0.72
	I_max_ %	<50	97.29 ± 1.61	<50	96.96 ± 5.75	<50	100	<50	100	<50	100	<50	98.49 ± 1.76	<50	100

Data represent mean I_max_ (the maximum observed inhibitory effect in %) and IC_50_ (µM) for MVH compounds when their antagonistic properties are evaluated against all ELR+ chemokines (i.e., CXCL1-3 and CXCL5-8) (n = 3).

**FIGURE 1:**
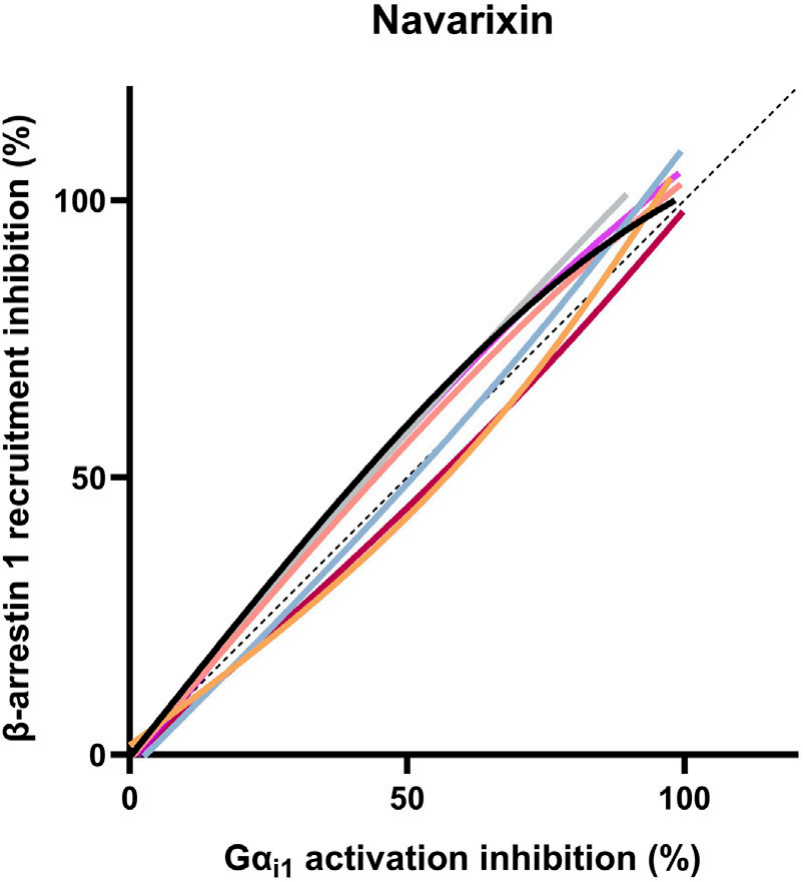
Bias plot of navarixin antagonism of ELR+ chemokine-induced CXCR2 signaling. An equimolar comparison between the different pathways using the mean of three independently measured concentration–response curves, where, for every pathway individually, the negative control is taken as 100% inhibition. The dotted line represents the theoretical trajectory of an unbiased profile.

It is clear from the data in Table 2 that navarixin showed comparable efficacy and potency in inhibiting Gα_i1_ activation and β-arrestin1 recruitment, irrespective of the ELR+ chemokine used. To further illustrate navarixin’s unbiased activity, a bias plot, which is an equimolar comparison plot in which the percentage inhibition of β-arrestin1 recruitment and Gα_i1_ dissociation for the different CXCR2 endogenous ligands is compared, was constructed (Figure 1). It is clear that navarixin inhibits ELR+ chemokine-induced CXCR2 β-arrestin1 recruitment and Gαi1 activation in an unbiased manner for all endogenous CXCR2 ligands, with only slight deviations from the theoretical unbiased compound profile (dotted line). Hence, navarixin was considered as a balanced reference compound for further analysis.

### Most MVH compounds are more potent in inhibiting ELR+ chemokine-induced CXCR2 β-arrestin1 recruitment than Gα_i1_ protein activation

To investigate the potential bias of the previously reported CXCR2 antagonists (Van Hoof et al., 2022), we applied both the Gα_i1_ protein dissociation and β-arrestin1 recruitment assays, as described before (Table 2). Among the evaluated compounds, **AZ10397767** and **MVH-23**, both thiazolo[4,5-*d*]pyrimidines carrying either an oxo or amino group at position 2, displayed similar efficacy as navarixin in inhibiting both pathways. Additionally, both the most potent compounds reported by the calcium mobilization assay (Van Hoof et al., 2022), that is, triazolo[4,5-*d*]pyrimidine **MVH-9** and isoxazolo[4,5-*d*]pyrimidine **MVH-24**, were able to inhibit G protein activation and β-arrestin1 recruitment. However, **MVH-9** and **MVH-24** were found to be more potent in β-arrestin1 recruitment inhibition (with IC_50_ values in the 0.03–0.17 µM range) than in Gα_i1_ activation (IC_50_ values in the 0.32–4.72 µM range). The other congeners were unable to inhibit Gα_i1_ activation, with IC_50_ values exceeding 2 µM, whereas they still displayed promising activity as inhibitors of β-arrestin1 recruitment, with IC_50_ values of less than 100 nM for most derivatives. For most compounds, inhibition of CXCR2 was dependent on the chemokine ligand used.

For example, **MVH-46** effectively inhibits CXCL2-, CXCL6-, and CXCL7-induced Gα_i1_ protein activation at low micromolar concentrations (IC_50_ values in the range of 2.6–3.5 µM). In contrast, when applying CXCL1 and CXCL8 as the agonists, **MVH-46** inhibits Gα_i1_ protein activation only at high micromolar concentrations (IC_50_ values 12.9–14.0 µM), and it is completely unable to inhibit CXCL3-mediated Gα_i1_ activation.

### Qualitative assessment using bias plot reveals the biased profile of MVH compounds toward β-arrestin1 recruitment inhibition over G protein activation, with navarixin used as a reference antagonist

**FIGURE 2:**
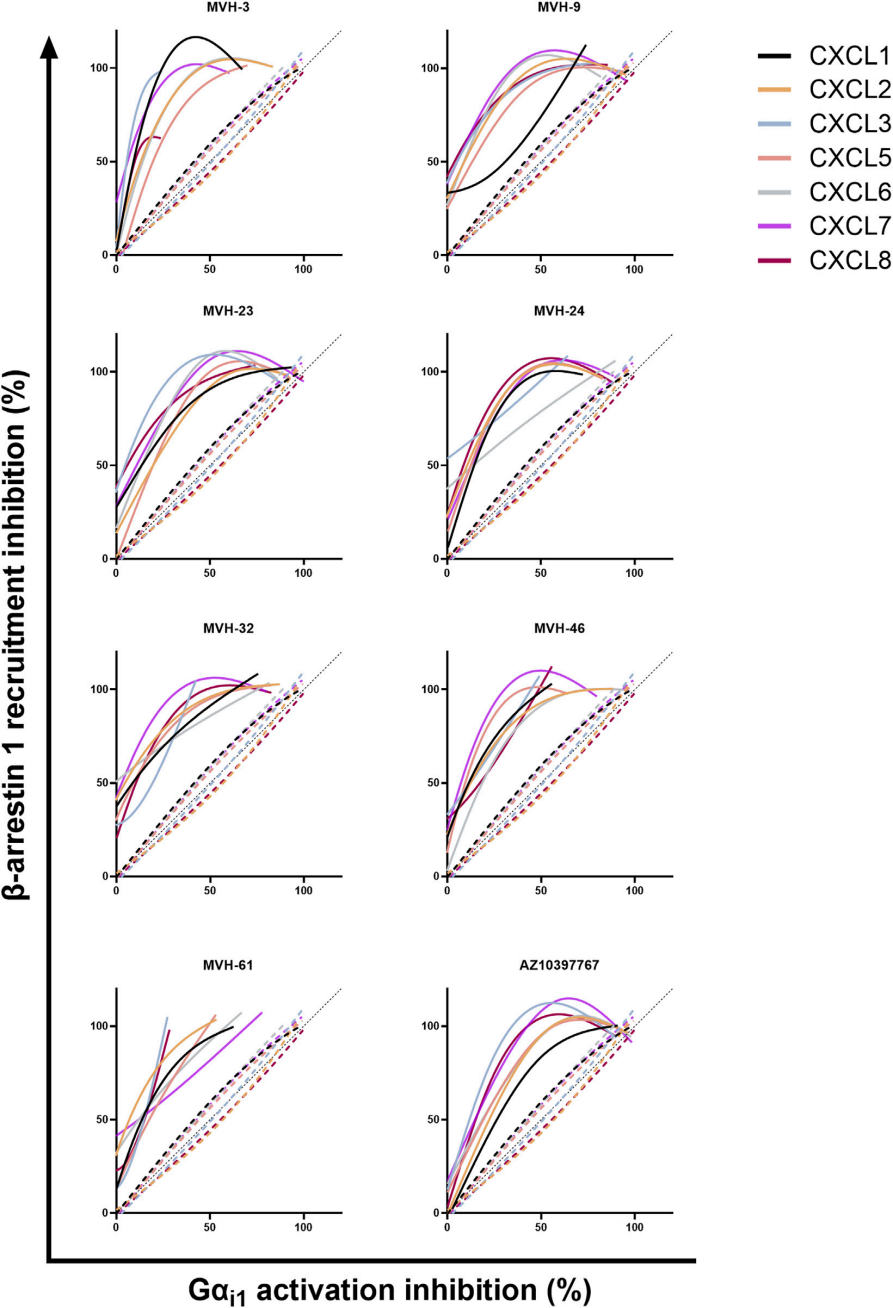
Bias plots of the compounds and navarixin for all seven CXCR2 chemokines. Data shown are the mean of three independent experiments. Dotted lines are navarixin data. Black dotted line *y* (*x*) = *x* exceeding (100,100) represents unbiased behavior. Color code for the used chemokines is identical to that used in Figure 1.

To further substantiate the potential biased activity of the MVH compounds, bias plots were generated for all compounds and compared with the antagonistic activity of navarixin (Figure 2). **AZ10397767** and all MVH compounds consistently exhibited a bias toward the inhibition of β-arrestin1 recruitment over the inhibition of G protein activation (Gα_i1_), following ELR+ chemokine-induced CXCR2 activation, compared to navarixin as the reference antagonist.

Notably, compounds **AZ10397767**, **MVH-9**, **MVH-23**, **MVH-24**, and **MVH-32**, while being biased toward β-arrestin1 inhibition, also demonstrated potent Gα_i1_ protein activation inhibition at higher concentrations across most ligands, indicating that their biased activity is concentration-dependent. The bias profile also depends on the chemokine ligand used. For instance, **MVH-9** exhibited a different inhibition profile for CXCL1-induced activation compared to other ELR+ chemokines. Similarly, **MVH-24** displayed distinct bias profiles for CXCL3 and CXCL6, whereas **MVH-32** showed a different bias profile for CXCL3-induced CXCR2 signaling inhibition. **MVH-46** completely inhibited CXCL2-and CXCL6-induced Gα_i1_ activation and β-arrestin1 recruitment at high concentrations, thus diminishing the biased profile at high concentrations. In contrast, when CXCL3 was applied as the ligand, **MVH-46** did not reach 50% inhibition of the Gα_i1_ protein activation pathway, maintaining a clear bias toward β-arrestin1 recruitment inhibition even at high concentrations.

### Quantitative assessment using bias index confirms bias toward the inhibition of β-arrestin1 recruitment compared to the inhibition of Gα_i1_ protein activation for all MVH compounds using navarixin as a reference

To quantitatively assess biased inhibition profiles, the I_max_ and IC_50_ parameters were used to calculate the bias index between the inhibition of the Gα_i1_ protein activation (P2) and β-arrestin1 recruitment (P1) pathways, relative to navarixin (**calculation 3**). Given the difficulty in fitting curves for compounds that did not reach 50% inhibition, we assigned a log(I_max_/IC_50_) of zero to these cases. A positive bias index indicates compounds biased toward inhibiting β-arrestin1 recruitment over Gα_i1_ activation using navarixin as the reference, with the absolute value indicating the extent of this bias.

**FIGURE 3:**
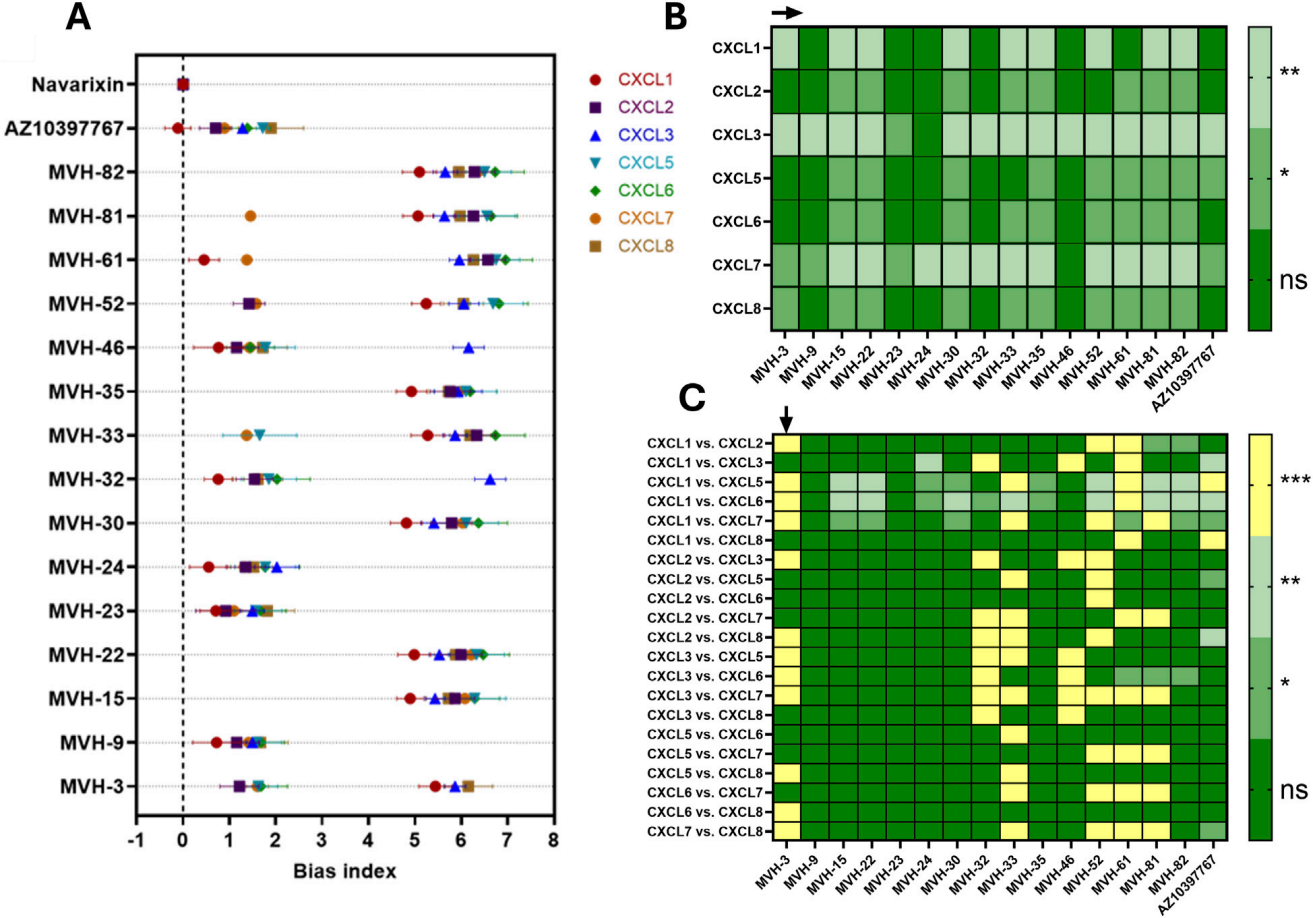
Bias index overview: **(A)** bias index between β-arrestin1 recruitment and Gα_i1_ protein activation inhibition was calculated for each compound using the different CXCR2 chemokines. Navarixin was used as the reference compound, with a bias index of zero. Data are presented as mean ± SEM, with a sample size of n = 3. **(B)** Statistical significance of H1 was assessed by comparing each compound to navarixin using a one-sample T-test with Benjamini–Hochberg correction applied for multiple comparisons. **(C)** Statistical significance of H2 was assessed by comparing ligand-induced responses per compound using a one-way ANOVA (α = 0.05), followed by Tukey multiple comparison test. Results are categorized as follows: p > 0.05 (ns), p < 0.01 (*), p < 0.001 (**), and p < 0.0001 (***).

The bias indexes shown in Figure 3A confirmed that the majority of the compounds displayed bias toward β-arrestin1 recruitment compared to Gα_i1_ protein activation using navarixin as the reference. Moreover, variations in the bias index across the different ligands were observed. In order to definitively claim “bias,” two hypotheses were raised. The first hypothesis (H1) investigated if the bias index of the MVH compounds is significantly different from navarixin within a single ligand. For this purpose, one sample T-tests were performed for each compound independently, and the corresponding p-values are shown in Figure 3B. Interestingly, compounds **MVH-9**, **MVH-23**, **MVH-24**, **MVH-32**, **MVH-46,** and **AZ10397767**, which are the most potent CXCR2 antagonists in the calcium mobilization assay (Van Hoof et al., 2022), were nonsignificantly different from navarixin, for CXCL1-, CXCL2-, CXCL5-, CXCL6-, and CXCL8-induced responses. Additionally, MVH3 is nonsignificantly different from navarixin for CXCL2, CXCL5, and CXCL6 responses while being highly significant for CXCL1-induced responses. The remaining compounds are statistically different from navarixin across all ligands, with few exceptions. Overall, the majority of compounds behaved differently from navarixin, except for some compounds in CXCL1- and CXCL2-induced responses.

The second hypothesis (H2) aims to investigate if the observed switches between high or low bias indexes—indicative of significantly biased or unbiased β-arrestin1 recruitment inhibition—depend on the CXCR2 ligand applied. For this purpose, a one-way ANOVA, followed by Tukey’s test, was performed. Based on the p-values (Figure 3C), the observed differences for compounds **MVH-3**, **MVH-32**, **MVH-33**, **MVH-46**, **MVH-52**, **MVH-61**, and **MVH-81** between the various CXCR2 ligands are statistically significant. Among these, **MVH-3**, **MVH-46**, and **MVH-61** exhibited potent calcium mobilization antagonism. Altogether, this quantitative analysis confirmed that CXCR2 antagonism of these MVH compounds is generally biased toward β-arrestin1 recruitment inhibition and varied depending on the ligand stimulating CXCR2.

## Discussion

CXCR2 antagonism is a promising therapeutic approach for the treatment of inflammatory diseases and cancer. An emerging trend in GPCR drug discovery is the development of small-molecule biased agonists and antagonists. This endeavor is grounded in studies on the opioid receptor field where the side effects of opioid agonists were suggested to originate from β-arrestin-driven effects (Kelly et al., 2023). Although the data have not been entirely reproducible, it has spurred significant interest within the GPCR field, including chemokine receptor drug discovery, to explore biased small modulators (Kelly et al., 2023; Grudzien et al., 2023). In this study, we therefore investigated the potential biased properties of a panel of previously reported CXCR2 antagonists. The community guidelines for the analysis of biased activity in terms of GPCR activity or inhibition emphasize the importance of both qualitative and quantitative assessments to accurately classify a compound as biased, along with the necessity to include an unbiased reference molecule for analysis purposes (Kolb et al., 2022). We applied both methods and included navarixin as an unbiased reference compound as it showed comparable antagonistic activity in inhibiting Gα_i1_ dissociation and β-arrestin1 recruitment. As stated in Table 2, several CXCR2 antagonists did not reach an IC_50_ (i.e., 50% inhibition) value for Gα_i1_ activation inhibition, which presented a limitation in the quantitative assessment of the compound bias. Therefore, an arbitrary log(I_max_/IC_50_) value of zero was assigned to those compounds (Kolb et al., 2022; Namkung et al., 2018), allowing to reflect the observed bias more accurately. However, this approach also significantly reduced the statistical power of the quantitative assessment as it tends to amplify the perceived bias, helping to explain the nonsignificant differences observed in qualitatively clear biased cases.

The inhibition preferences between ELR+ -chemokine-induced G protein and β-arrestin activation was investigated using NanoBRET-based assays. Remarkably, most compounds were unable to achieve 50% inhibition of Gα_i1_ activation, even at the highest tested concentration of 15 μM; however, they effectively inhibited β-arrestin1 recruitment (Table 2). Although both assays employed the NanoBRET technology, differences in assay design may have contributed to the observed discrepancies, suggesting a potential observational or system bias rather than true signaling bias. Specifically, the β-arrestin1 recruitment assay involved NLuc-tagged CXCR2 co-transfection, which might result in lower CXCR2 expression levels than the stably transfected high-expression CXCR2 cell line used in the G protein assay. This lower expression might facilitate inhibition of CXCR2 signaling, leading to varying efficacies between the assays. However, navarixin, a potent CXCR1/2 antagonist, effectively inhibited both pathways, thus confirming the reliability of the assays and minimizing concerns related to the experimental setup. Additionally, in both assays, CXCR2 was stimulated with the different ELR+ chemokines at their corresponding EC_80_ concentrations to enable potency comparisons. The EC_80_ values of the ELR+ chemokines in the β-arrestin recruitment assay were consistently higher than those for G protein activation (Table 2). Despite this higher ligand concentration, the MVH compounds were substantially more potent in the β-arrestin recruitment assay.

Interestingly, the bias toward β-arrestin1 recruitment inhibition was observed for all compounds across all chemokine ligands. This is not unexpected as the MVH compounds are all structurally similar and derived from a single lead molecule. To our surprise, the observed bias was dependent on the specific CXCR2 ligand. This inhibition profile is particularly intriguing and appealing given that CXCR2-associated cancers are often characterized by the upregulation of one or two CXCR2 ligands (Nagarsheth et al., 2017; Zhang et al., 2020). Therefore, CXCR2 antagonists that selectively inhibit downstream pathways in a ligand-dependent manner could offer a strategic advantage. Such ligand-dependent biased antagonism could enable more targeted modulation of context-dependent “pathological signaling” while potentially preserving other physiological chemokine functions compared to ligand-independent balanced antagonists.

Importantly, in this study, only one subtype of each transducer family was selected, Gα_i1_ and β-arrestin1. These were chosen as they were previously shown to be the most effectively activated by CXCR2 (17). However, CXCR2 activates other subsets as well, such as Gα_i2,_ Gα_i3,_ Gα_oA,_ Gα_oB_, Gα_15_, and β_2_ (Boon et al., 2024). Hence, it is worthwhile to investigate if the β-arrestin1 bias still exists when using the other subtypes.

## Conclusion

In this study, a series of previously discovered intracellular allosteric CXCR2 antagonists was characterized for their biased properties. NanoBRET-based assays were used to investigate the biased activity of the MVH compounds, with navarixin serving as a balanced reference. It was discovered that these CXCR2 antagonists demonstrated a consistent preference for β-arrestin1 recruitment inhibition over the inhibition of Gα_i1_ protein activation. Moreover, the bias profiles varied depending on the specific ELR+ chemokine activating CXCR2, highlighting a ligand-dependent antagonistic effect. Although there are no proven benefits to biased antagonism of hGPCRs, here, we present a workflow that can be used to investigate biased antagonism between G proteins activation and β-arrestin recruitment.

## Data Availability

The original contributions presented in the study are included in the article/Supplementary Material; further inquiries can be directed to the corresponding author.
