# Supplementary material for: Biased antagonism of a series of bicyclic CXCR2 intracellular allosteric modulators
Source: Front Pharmacol. 2025 Jul 14;16:1631129. doi: 10.3389/fphar.2025.1631129 (PMC12301332; doi:10.3389/fphar.2025.1631129)
Supplement: Supplementary file 1 [file Table1.docx]

**Table S1. Overview of the compound library created by Dr. Max Van Hoof ^5,14^**

| **Compound** | **Structure** | **Molecular weight (g/mol)** | **Source** | |  |
| --- | --- | --- | --- | --- | --- |
| **AZ-10397767** | 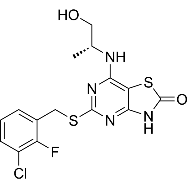 | 400.88 | MedChem  -Express | |  |
| **NAVARIXIN** | 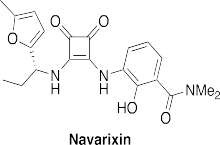 | 397.43 | MedChem  -Express | |  |
| **AZD-8309** | 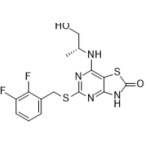 | 384.42 | MedChem  -Express | |  |
| **MVH-3** | 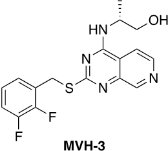 | 362.4 | Dr. M. Van Hoof | |  |
| **MVH-9** | **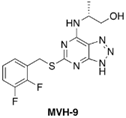** | 352.36 | Dr. M. Van Hoof |  |  |
| **MVH-15** | 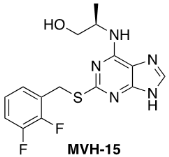 | 351.38 | Dr. M. Van Hoof | |  |
| **MVH-22** | 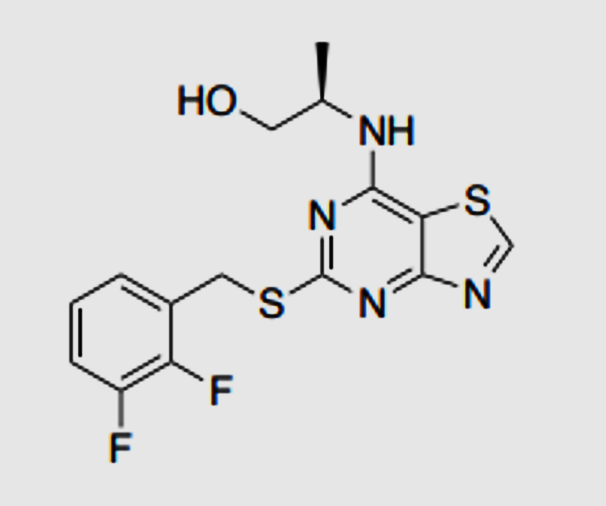 | 368.42 | Dr. M. Van Hoof | |  |
| **MVH-23** | 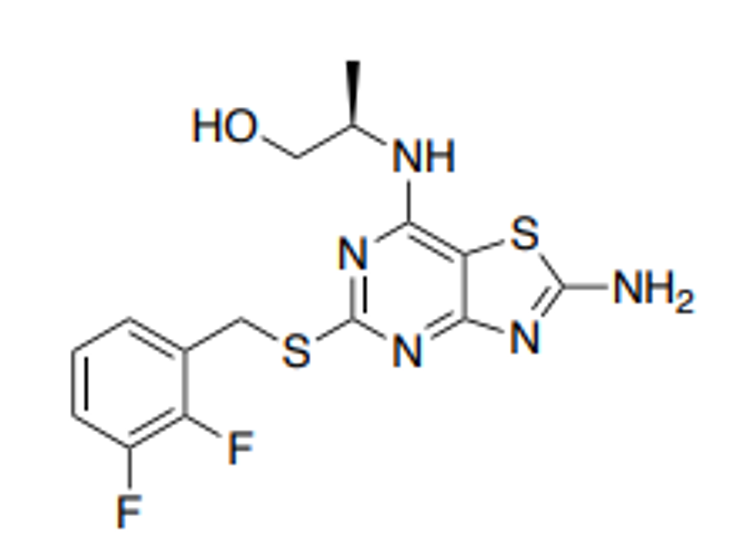 | 383.44 | Dr. M. Van Hoof | |  |
| **MVH-24** | 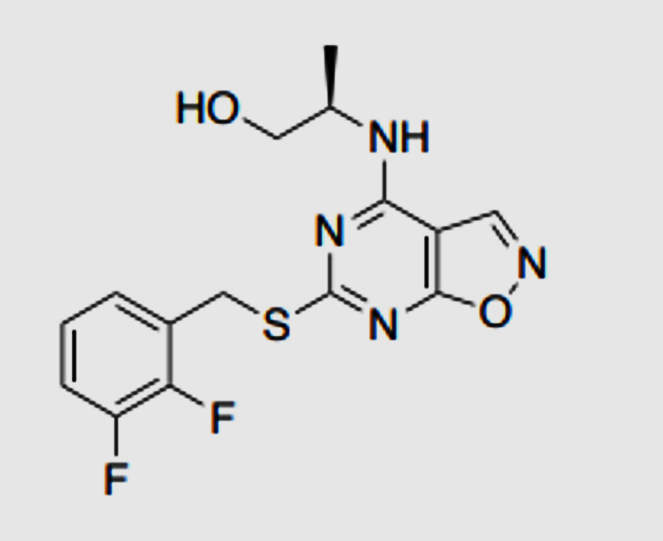 | 352.38 | Dr. M. Van Hoof | |  |
| **MVH-30** | 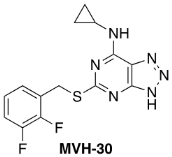 | 334.35 | Dr. M. Van Hoof | |  |
| **MVH-32** | 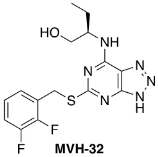 | 366.39 | Dr. M. Van Hoof | |  |
| **MVH-33** | 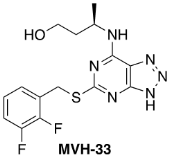 | 366.39 | Dr. M. Van Hoof | |  |
| **MVH-35** | 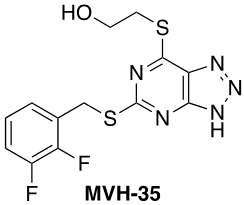 | 355.38 | Dr. M. Van Hoof | |  |
| **MVH-46** | 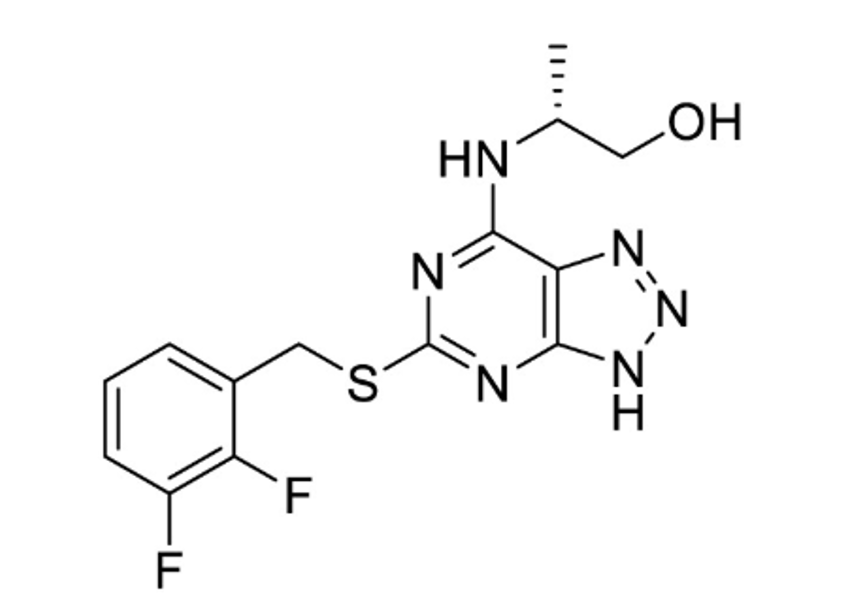 | 366.36 | Dr. M. Van Hoof | |  |
| **MVH-52** |  | 336.3 | Dr. M. Van Hoof | |  |
| **MVH-61** |  | 322.43 | Dr. M. Van Hoof | |  |
| **MVH-81** |  | 294.38 | Dr. M. Van Hoof | |  |
| **MVH-82** | 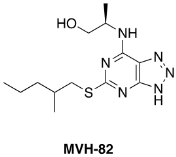 | 310.42 | Dr. M. Van Hoof | |  |
