# Supplementary figures and images for: Biased antagonism of a series of bicyclic CXCR2 intracellular allosteric modulators
Source: Front Pharmacol. 2025 Jul 14;16:1631129. doi: 10.3389/fphar.2025.1631129 (PMC12301332; doi:10.3389/fphar.2025.1631129)

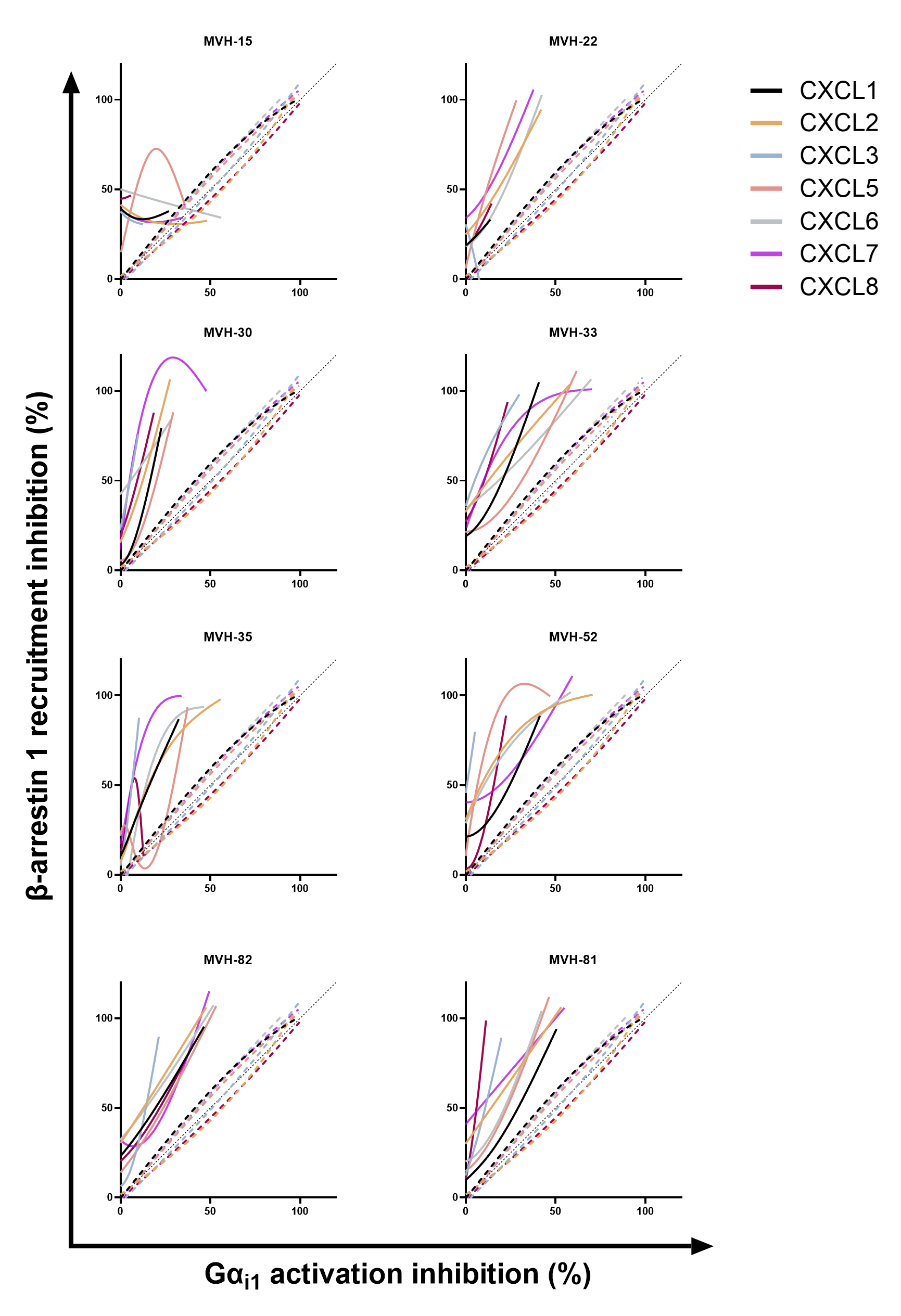

Supplement: Supplementary file 2 [file Image1.tif]
